# Artificial Intelligence in Spine Surgery: Imaging-Based Applications for Diagnosis and Surgical Techniques

**DOI:** 10.1007/s12178-025-09972-9

**Published:** 2025-04-30

**Authors:** James S. MacLeod, Tyler Compton, Yianni Bakaes, Avani Chopra, Frances Akwuole, Cole Christenson, Wellington Hsu

**Affiliations:** 1 Department of Orthopaedic Surgery, Northwestern University, 259 E Erie St, Chicago, IL 60611 USA; 2 Center for Regenerative Nanomedicine, Northwestern University, Chicago, IL USA

**Keywords:** Artificial intelligence, Spine surgery, Imaging, Oncology, Surgical navigation, Robotics

## Abstract

**Purpose of Review:**

Artificial intelligence (AI) has rapidly proliferated though medicine with many novel applications to improve patient care and optimize healthcare delivery. This review investigates recent literature surrounding the influence of AI imaging technologies on spine surgical practice and diagnosis.

**Recent Findings:**

Robotic-assisted pedicle screw placement has been shown to increase the rate of clinically acceptable screw placement while increasing operative time. AI technologies have also shown promise in creating 3D spine imaging while reducing patient radiation exposure. Several models using various imaging modalities have been shown to reliably identify vertebral osteoporotic fractures, stenosis and spine cancers.

**Summary:**

Complex spinal anatomy and pathology as well as integration of robotics make spine surgery a promising field for the deployment of AI-based imaging technologies. Imaging-based AI projects show potential to enhance diagnostic and surgical efficiency, facilitate trainee learning and improve operative outcomes.

## Introduction

The field of spine surgery has continually evolved alongside advancements in imaging technology which enhance the ability of surgeons to diagnose spine pathology, develop preoperative plans, and perform complex procedures with greater precision. Emerging technologies leverage radiographs (X-ray), ultrasound, computerized tomography (CT), and magnetic resonance imaging (MRI), providing higher-resolution images, improved tissue differentiation, and expanded diagnostic capabilities. Recently, there have been significant advancements made in the realm of imaging resulting in reduced radiation exposure, improved image acquisition, and new applications of imaging contributing to a paradigm shift toward minimally-invasive techniques and patient-specific surgical strategies.

As imaging technology continues to advance, artificial intelligence (AI) has emerged as a transformative force, offering the potential to complement existing imaging modalities. AI excels in image optimization, as a diagnostic aid, and in operative visualization and planning. AI-powered algorithms, including deep learning and computer vision, can identify patterns and anomalies that may elude human observation, enabling earlier diagnosis, risk minimization, and personalized treatment planning [1].

In the realm of spine surgery, AI applications are rapidly expanding, with promising contributions in areas such as automated detection of spinal pathology, segmentation of intervertebral discs, prediction of surgical outcomes, and intraoperative navigation. For example, AI is already shown to be proficient at classifying degenerative discs according to Pfirrmann grading (a category ranging from 1 [healthy disc] to 5 [severe disc degeneration]) via feature-extraction from MRI images [2, 3]. AI-driven image analysis has shown promise in reducing variability in preoperative planning and guiding real-time adjustments during procedures [4, 5]. The applications of AI in spine imaging are vast and a recent review has covered AI based imaging enhancement modalities and anatomical landmark guided diagnosis aids [6].

### Applications in Robotic Spine Surgery

**Fig. 1:**
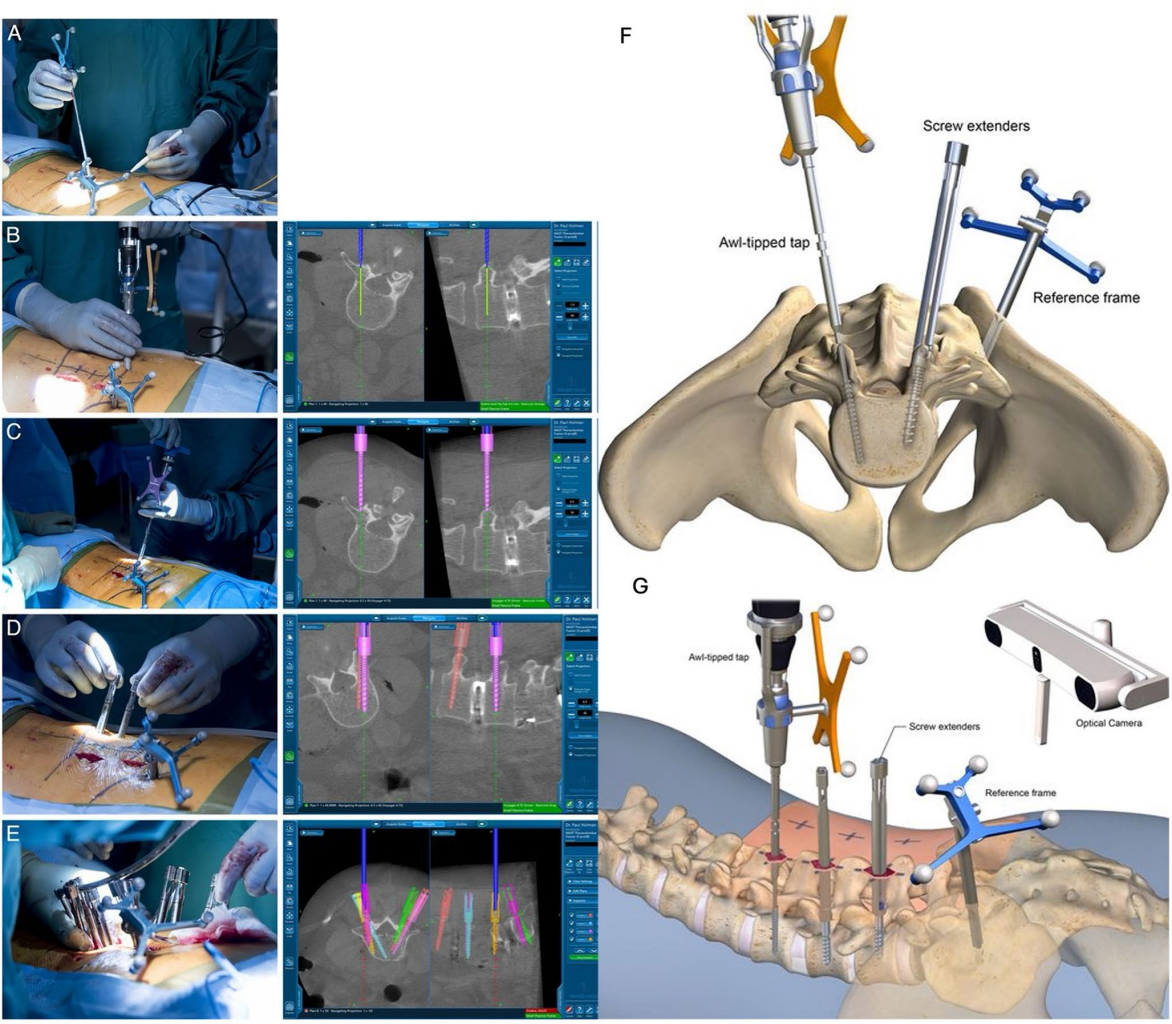
(**A**) Navigation technology is used to optimize the incision placement for minimally invasive spine surgery. (**B**, **C**, **D** and **E**) Steps of the guided pedicle placement procedure are visualized along with corresponding imaging. (**F** and **G**) Illustrations showing the AI guided pedicle screw placement system and operative set up. Included with permission from the International Journal of Spine Surgery. All figures labeled for reuse according to Creative Commons Non-Commercial-No-Derivatives 4.0 [10]

Robotic technologies, particularly in the context of pedicle screw placement, have demonstrated significant advancements in surgical precision and safety. Various imaging technologies, such as ultrasound and CT, have been integrated to support pedicle screw placement and intraoperative navigation [7]. Robotic systems have been associated with higher rates of clinically acceptable pedicle screw placement compared to traditional freehand techniques, Fig. 1(A-G) [4, 5, 8–10]. Fatima et al. reported the rate of perfect and clinically acceptable pedicle screw accuracy (defined by Gerztbein-Robbin Grade A + B) was significantly increased with robotic-assisted surgery compared to free-hand technique. However, they also reported significantly longer operative times in the robotic group, a common finding [5]. This trade-off underscores the importance of balancing precision with efficiency, especially in cases where time is a critical factor. Robotic spine surgery presents an interesting use-case for the application of AI in spine surgery. AI may be able to make robotic spine surgery more time efficient and decrease radiation exposure to patients. AI-driven 3D reconstructions, similar to CT scans, can be generated from fluoroscopy and MRI images. These reconstructions offer comparable accuracy to fluoroscopy-guided freehand techniques and CT-guided methods while reducing radiation exposure and, in some cases, providing greater anatomical detail [11–13]. These applications can also personalize screw placement to minimize the chance of screw pull-out, which is especially useful in osteoporotic patients [14].

**Fig. 2:**
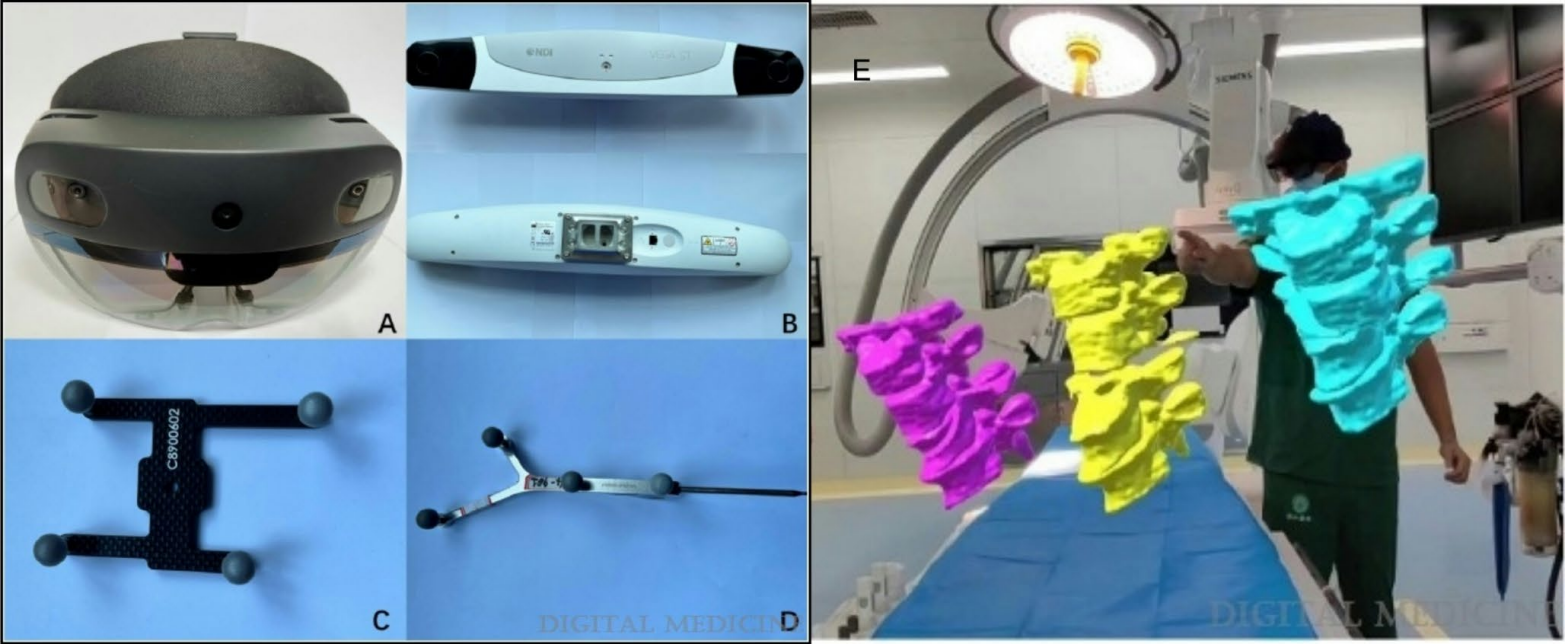
Example of an augmented reality (AR) surgical navigation system. (**A**) AR headset (Microsoft HoloLens). (**B**) Surgical localization system (Northern Digital Inc.) which tracks surgical instruments in the operative field. (**C** and **D**) Example of calibration tools placed on operative instruments or at certain anatomical locations to orient the system. (**E**) Learners using AR technology to prepare for a spine surgery case. This technology can be used preoperatively or intraoperatively to better understand relevant anatomy, and surgical planning. Included with permission from Wolters Kluwer Health, Inc. All figures labeled for reuse according to Creative Commons Non-Commercial-No-Derivatives 4.0 [52]

In addition to lengthened operative times, robot-assisted surgery introduces a notable learning curve for surgeons, an obstacle that AI may be able to mitigate through the use of augmented reality (AR) and virtual reality (VR) outside and inside of the operating room, Fig. 2 [15]. Proper training and hands-on experience are necessary to maximize the benefits of these technologies while minimizing complications. Studies have highlighted the importance of preoperative education, simulation training, and real-world experience to reduce errors during the adoption phase [15, 16]. Addressing the learning curve through structured training programs may help accelerate the integration of robotic systems into standard surgical practice, improving outcomes while mitigating the drawbacks of longer operative times. Intraoperatively, AR-assisted pedicle screw placement combines digital overlays with real-time anatomical visualization [17]. This technology has demonstrated accurate screw placement in cadaveric and clinical studies through real-time visualization, suggesting its potential as a viable training modality for surgeons in the early stages of their careers, those transitioning to new systems and as an adjunct for experienced surgeons [18–23].

### Complication Prevention

**Fig. 3:**
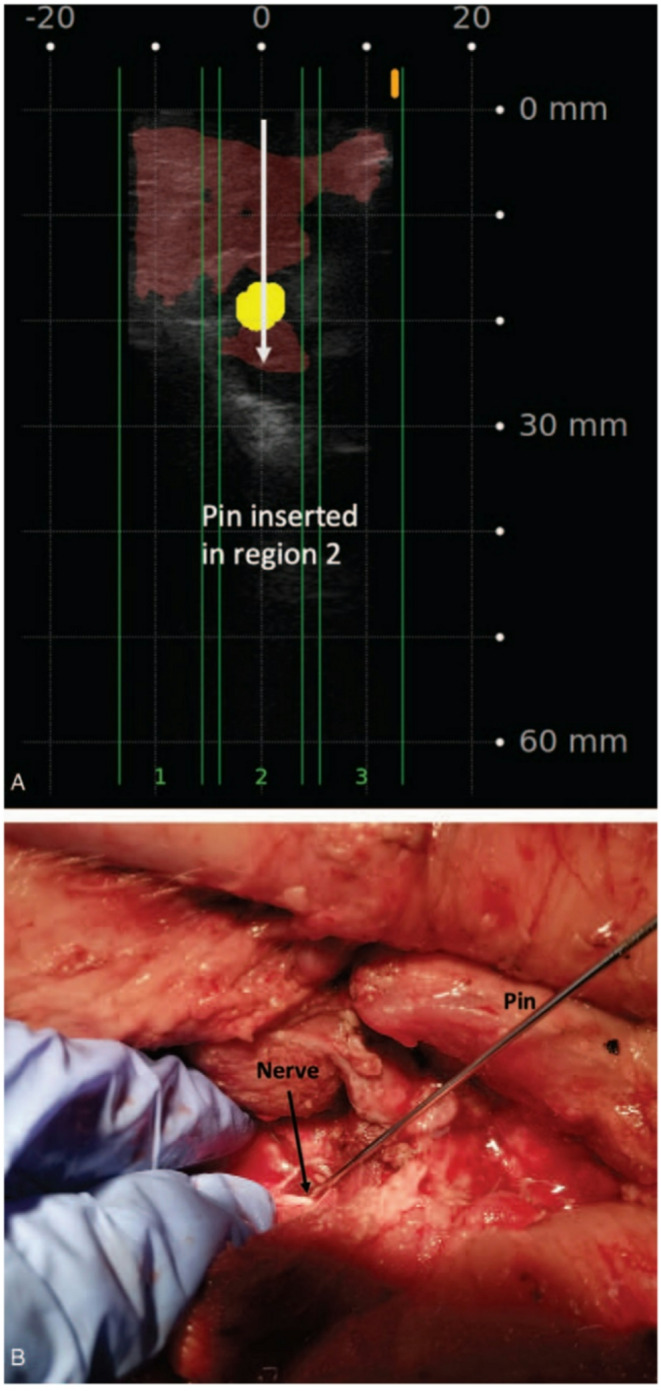
(**A**) SonoVision display showing a nerve region (highlighted in yellow) located about 20 mm deep within the muscle. (**B**) Surgical dissection confirming the nerve identified in (**A**). Included with permission from Wolters Kluwer Health, Inc. All figures labeled for reuse according to Creative Commons Non-Commercial-No-Derivatives 4.0 [19]

Another application of AI in spine surgery involves complication prevention. It can sometimes be difficult to identify the intended operative level and pertinent anatomical structures due to anatomical variations and the minimally invasive nature of many procedures. Mody et al. reported the rate of wrong-level surgery to be 1 in every 3110 spinal fusion procedures and that 50% of spine surgeons have performed a wrong-level surgery during their career [24]. Notably, such cases result in litigation at rates ranging from 17 to 99%, and often lead to additional procedures [24, 25]. AI vertebral level identification has been shown to be effective using both CT and fluoroscopic images [26, 27]. An AI powered ultrasound platform has shown promise in identifying anatomical structures and differentiating between nerve, muscle, bone and other structures intraoperatively, Fig. 3 [19]. Leveraging the strengths of imaging modalities with AI-based image analysis can further enhance the accuracy and safety of fusion and other procedures by preventing wrong level surgery, damage to nearby structures and by optimizing pedicle screw placement, creating opportunity for hybrid systems that combine multiple imaging sources to optimize outcomes.

In summary, studies have demonstrated that robotic pedicle screw placement provides greater precision compared to traditional techniques, though further research is needed to evaluate long-term clinical benefit. AI may be able to improve operative times and usability of these robotic systems in the future. Additional applications of AI in spine surgery could include the development of machine learning models capable of tracking surgical steps and alerting surgeons to potential errors or dangers including preventing wrong level procedures and damage to nearby structures. AI-powered training can also be implemented to flatten the learning curve of robotic surgery and increase trainee accuracy. These innovations hold the potential to further enhance surgical safety and precision, ensuring optimal outcomes for patients undergoing spine surgery.

### Osteoporotic Fracture Detection, Traumatic Fractures and Stenosis

AI imaging applications have shown remarkable potential for the assessment and diagnosis of various spine pathologies. Machine learning algorithms, particularly deep learning models, have been successfully used to analyze radiographs for the detection of osteoporotic vertebral compression fractures, achieving diagnostic accuracy comparable to that of experienced radiologists [28, 29]. AI applications have shown promise in being an adjunct to radiologists and surgeons with a recent study finding AI-assisted human detection to be superior to human detection and AI detection [30]. Another example is AI_OVF_SH (artificial intelligence_ osteoporotic vertebral fracture_Sixth People’s Hospital), a deep-learning-based osteoporotic vertebral fracture diagnostic system which was shown to reduce the rate of missed diagnoses, and reduce physician workload [31]. In this study, radiologists demonstrated a sensitivity of 54.11% in detecting osteoporotic vertebral fractures without assistance from an intelligent diagnostic system, highlighting a significant underdiagnosis rate. In comparison, the AI_OVF_SH system achieved a sensitivity of 83.35% with a speed of 41 frames per second [31].

Beyond radiographs, machine learning models have also been developed for the analysis of osteoporotic vertebral fractures on CT. These CT-based algorithms leverage the superior anatomical detail to identify subtle fractures that might otherwise be missed, particularly in difficult cases where the anatomy is distorted by prior surgery, or there is severe osteoporosis [32–34]. An example is Tomita et al. who reported detection of osteoporotic vertebral fractures with 89.2% accuracy on CT, matching the performance of practicing radiologists [34]. Similarly, AI applications in MRI have been utilized not only for fracture detection but also for assessing the risk of non-union in osteoporotic vertebral fractures, providing critical prognostic information that can guide clinical decision-making [35–37].

**Fig. 4:**
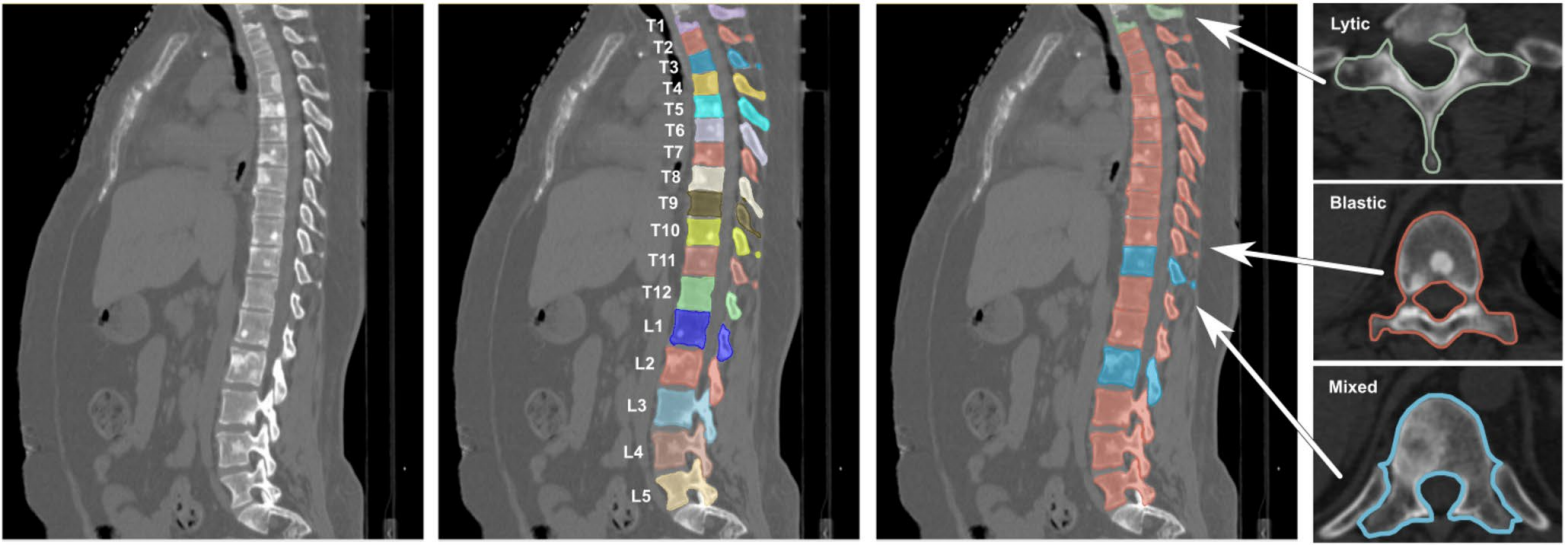
Example of artificial intelligence guided computed tomography (CT) spine segmentation and analysis of lytic, blastic, and mixed lesions. All figures labeled for reuse according to Creative Commons Non-Commercial-No-Derivatives 4.0 [53]

The use of AI diagnostic imaging applications extends beyond osteoporotic vertebral fractures and also includes the accurate diagnosis of traumatic thoracolumbar fractures and lumbar stenosis. Deep learning models have demonstrated high sensitivity and specificity in these contexts [38, 39]. Additionally, several studies have outlined the utility of machine learning models in the diagnosis, differentiation, and segmentation of spinal malignancy, Fig. 4 [36, 40–42].

### Oncology

A recent systematic review of oncologic applications of AI in CT spine imaging found 7 clinical studies (21.2%) that used machine learning to distinguish between benign and malignant lesions, 3 (9.1%) that evaluated tumor stage or grade, and 2 (6.1%) that employed radiomics for biomarker classification [43]. AI-based algorithms have been shown to detect metastatic lesions resulting in fracture and cord compression in CT scans with accuracy comparable to experienced radiologists [44–46]. Machine learning and deep learning models have also successfully discriminated between benign and pathologic bone marrow patterns on lumbar spine MRIs which can help diagnose hematologic disease, especially for less experienced clinicians [47]. Vertebral benign and malignant fractures were diagnosed with 92% accuracy by the ResNet50 model (deep learning model using convolutional neural networks), 98% by a senior musculoskeletal radiologist, 96% by a fourth year radiology resident and 66% by a first year radiology resident. Interestingly, the performance of the first year resident increased to 94% with the aid of the AI model, demonstrating the utility of these platforms for junior trainees [48].

Despite their promise, many AI-driven imaging applications have demonstrated high internal validity but face limitations in external validation [49, 50]. Models have been shown to struggle with tasks related to certain spinal pathology, for example sclerotic lesion segmentation [51] and are often trained repeatedly on specific datasets that may not account for variability in patient demographics, imaging protocols, or disease presentations [50]. This limited generalizability highlights the need for further research to continually validate these models using larger, more diverse datasets, ensuring robustness and mitigating potential biases present in smaller, less diverse datasets.

In summary, several validated AI models have been developed for the diagnosis of spinal pathologies, including osteoporotic fractures, traumatic injuries, stenosis, and spine tumors using multiple imaging modalities. These models have occasionally surpassed the diagnostic accuracy of radiologists and spine surgeons, highlighting their potential to complement clinical expertise. However, to ensure safety, reliability, and broad applicability, future research must focus on external validation, addressing scope limitations, and refining these technologies for diverse clinical environments.

## Conclusion

AI-driven imaging technologies are transforming spine surgery by enhancing diagnostic accuracy, surgical precision, and patient safety. While innovations such as AI-assisted robotic pedicle screw placement and deep learning-based pathology detection have demonstrated promising results, challenges remain in balancing efficiency with precision and ensuring external validation for widespread adoption. Future research should focus on refining machine learning models, improving training methodologies, and integrating multimodal imaging systems to optimize clinical outcomes. As AI continues to evolve, its thoughtful implementation will be crucial in driving the next era of innovation in spine surgery.

## Key References

Han, H., et al., *Revolutionizing spinal interventions: a systematic review of artificial intelligence technology applications in contemporary surgery.* BMC Surgery, 2024. **24**(1): p. 345.

This systematic review covers the broad range of AI applications in spine surgery. It includes 90 articles focusing on AI’s role for assessing surgical indications, assisting in surgical procedures, predicting surgical outcomes and predicting complications in spine surgery. The article emphasizes AI’s role as a supplement and the authors do not predict human surgeons being replaced by these technologies in the foreseeable future.

Luchmann, D., et al., *Spinal navigation with AI-driven 3D-reconstruction of fluoroscopy images: an ex-vivo feasibility study.* BMC Musculoskelet Disord, 2024. **25**(1): p. 925.

This feasibility study assessed an AI-based program that creates a three dimensional anatomical model of the spine from fluoroscopy images. This technology offers potential for cost saving and decreased radiation exposure. Surgeons guided by the program’s reconstructed models showed non-inferiority to the fluoroscopy guided free hand control group in an ex-vivo setting, prompting further testing in operative settings.

Liawrungrueang, W., et al., *Osteoporotic vertebral compression fracture (OVCF) detection using artificial neural networks model based on the AO spine-DGOU osteoporotic fracture classification system.* N Am Spine Soc J, 2024. **19**: p. 100,515.

This study used an artifical neural networks model to classify osteoportic vertebral compression fractures based on the AO Spine-DGOU classification system. The model was 96.04% accurate and the authors concluded that the model was both rapid and effective demonstating a use case for this technology for classifying these common fractures.

## Data Availability

No datasets were generated or analysed during the current study.
